# Supplementary material for: Lung Cancer: Spectral and Numerical Differentiation among Benign and Malignant Pleural Effusions Based on the Surface-Enhanced Raman Spectroscopy
Source: Biomedicines. 2022 Apr 25;10(5):993. doi: 10.3390/biomedicines10050993 (PMC9138770; doi:10.3390/biomedicines10050993)
Supplement: Supplementary file 1 [file biomedicines-10-00993-s001.zip › biomedicines-1663603-SM-xml.pdf]

Supplementary Material

Table S1. Sample classification based onto histopathological and immunohistochemical staining.

Causes	Disease	Number of Patients
Cancerous	adenocarcinoma	11
	squamous cell lung cancer	2
	breast cancer	1
	**Total**	14
Non-cancerous	embolism	1
	POChP	1
	heart failure	1
	other, non-cancerous	3
	**Total**	6

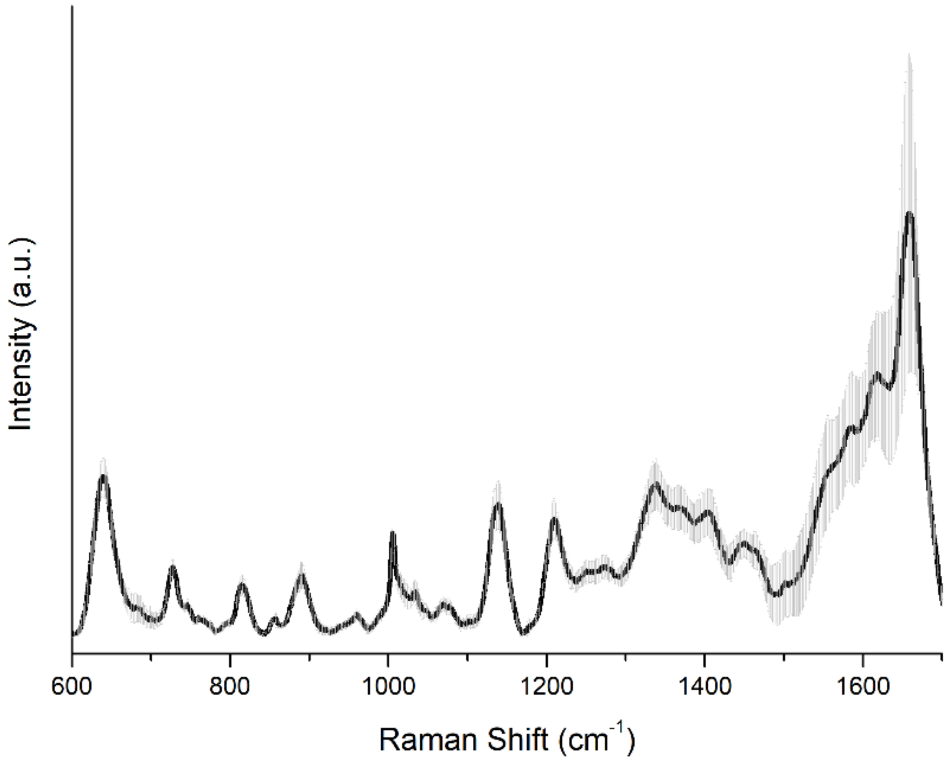

Figure S1. SERS spectra averages from the SERS map of adenocarcinoma pleural effusion, are based onto 15 spectra. The standard deviation on the average plots is visualized by the grey colour.

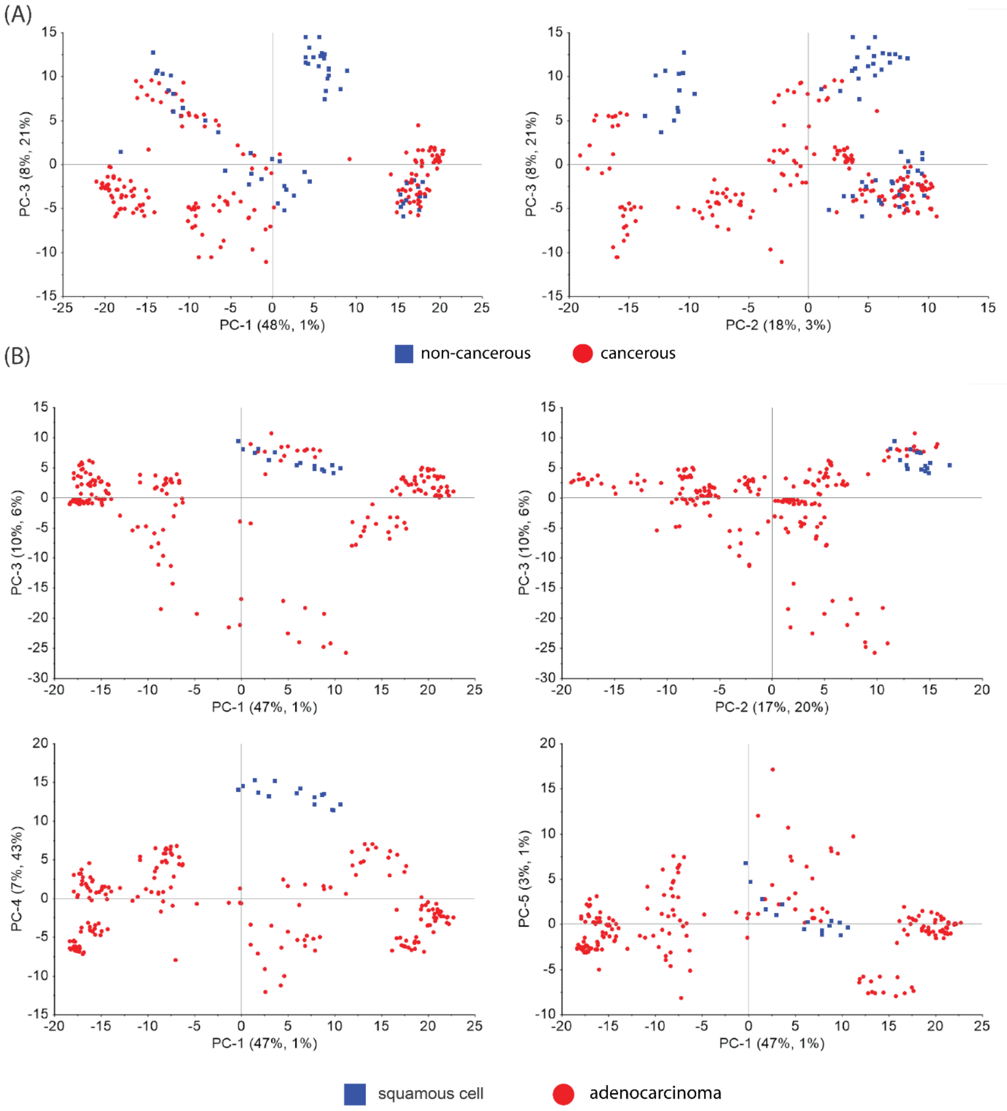

Figure S2. PCR scores plots of PC1 vs. PC3, PC2 vs. PC3 for non-cancerous and cancerous (**A**) and PC1 vs. PC3, PC2 vs. PC3, PC1 vs. PC4 and PC1 vs. PC5 for squamous cell and adenocarcinoma samples (**B**).

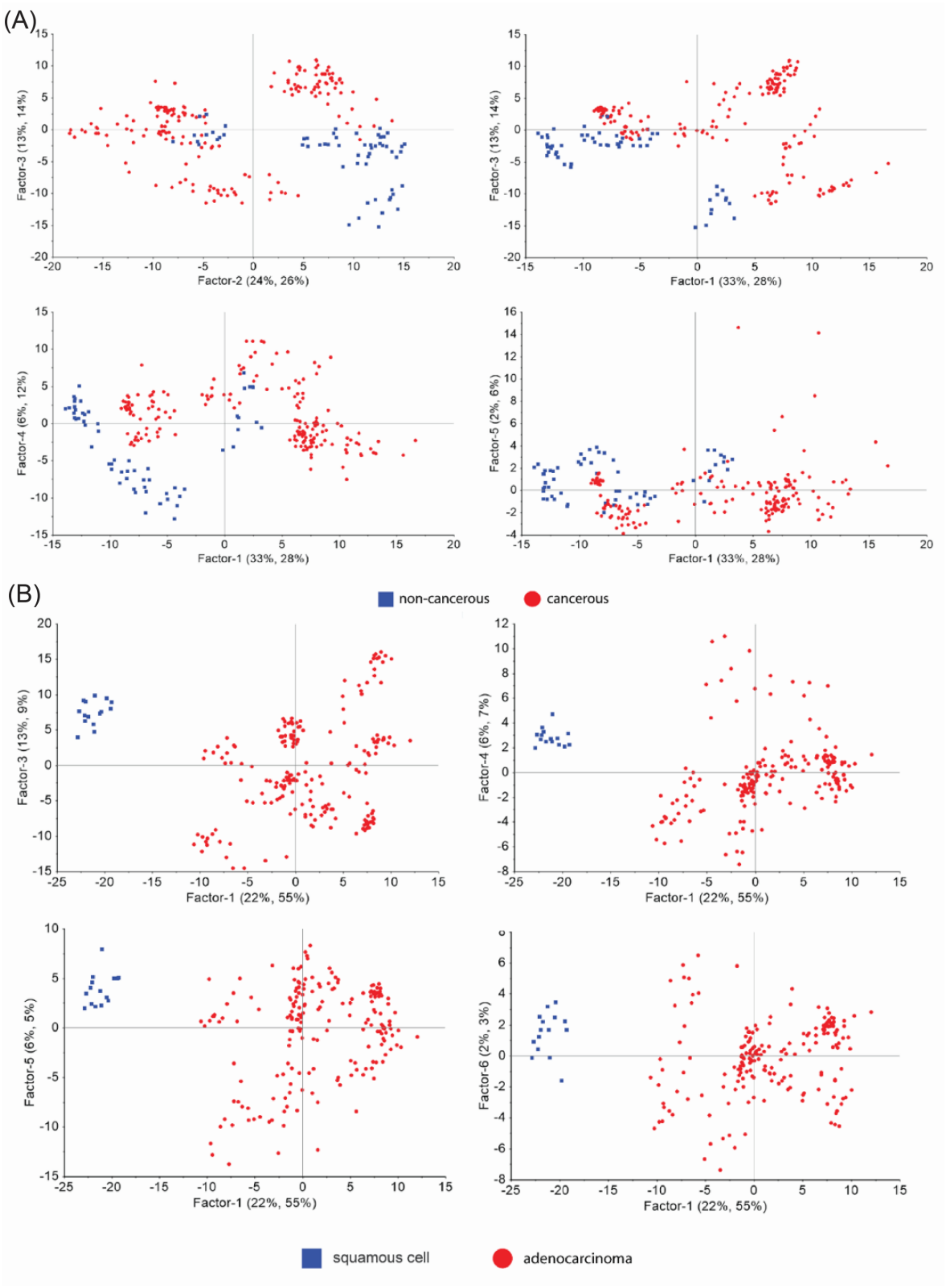

Figure S3. PLS scores plots of F-2 vs. F-3, F-1 vs. F-3, F-1 vs. F-4 and F-1 vs. F-5 for healthy and cancer (**A**) and for squamous and adenocarcinoma samples (**B**).

Table S2. The RMSE and R2 along with number of important PCs or Fs and their corresponding percentage values data obtained from PCR and PLS multivariate analysis.

PCR	RMSEC	RMSEP	R2C	R2P
Non-cancerous vs. Cancerous (13 PCs; 96 %)	0.1958	0.2058	0.7968	0.7770
Squamous vs. adenocarcinoma (4 PCs; 81%)	0.1390	0.1443	0.7089	0.6884

**PLS**				
Non-cancerous vs. Cancerous (10 Fs; 95%)	0.0901	0.1289	0.9570	0.9129
Squamous vs. adenocarcinoma (8 Fs; 95%)	0.0553	0.0069	0.9539	0.8964

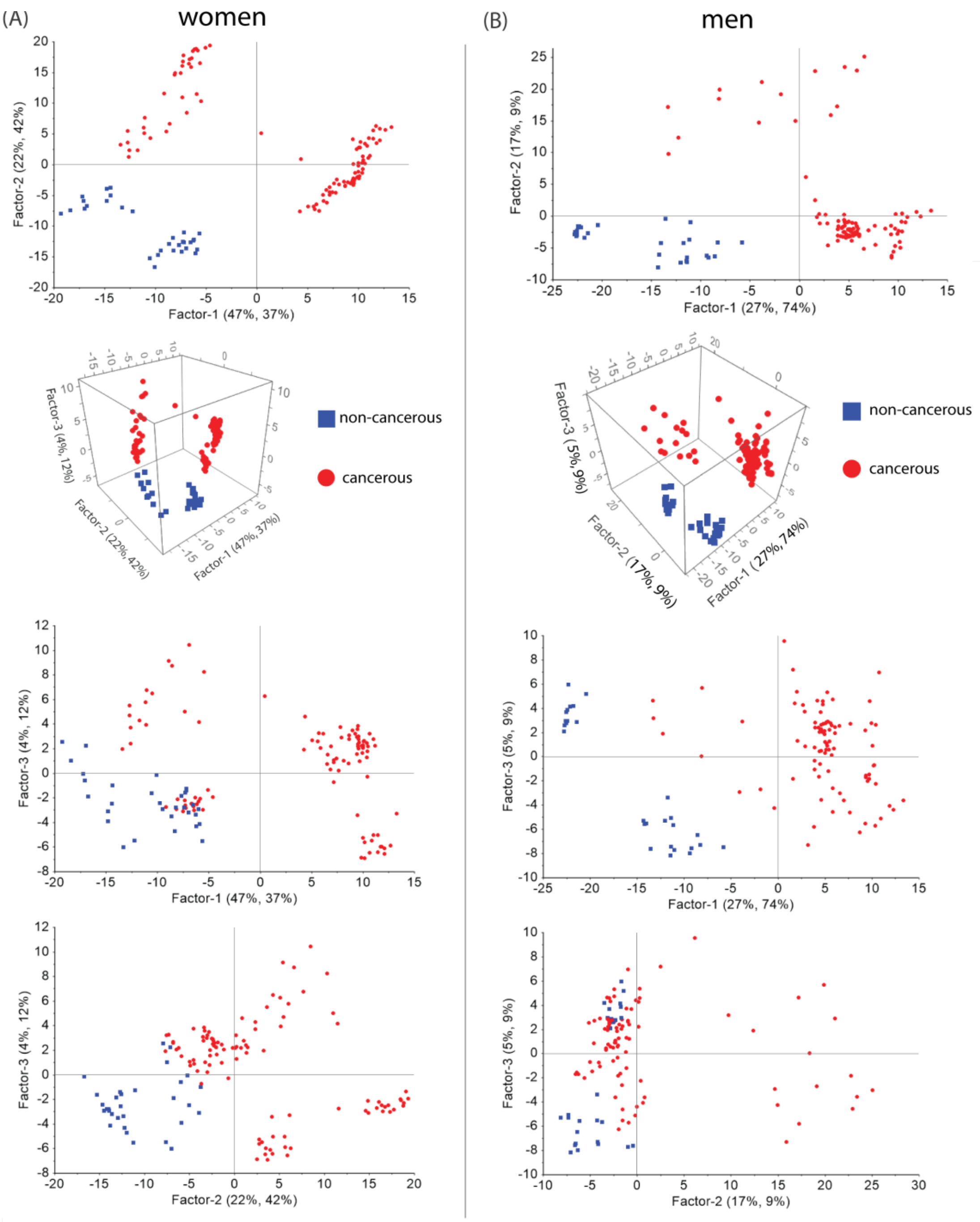

Figure S4. PLS scores plots of F-1 vs. F-2, F-1 vs. F-3 and F-2 vs. F-3 for non-cancerous and cancerous samples for women (**A**) and for men (**B**).

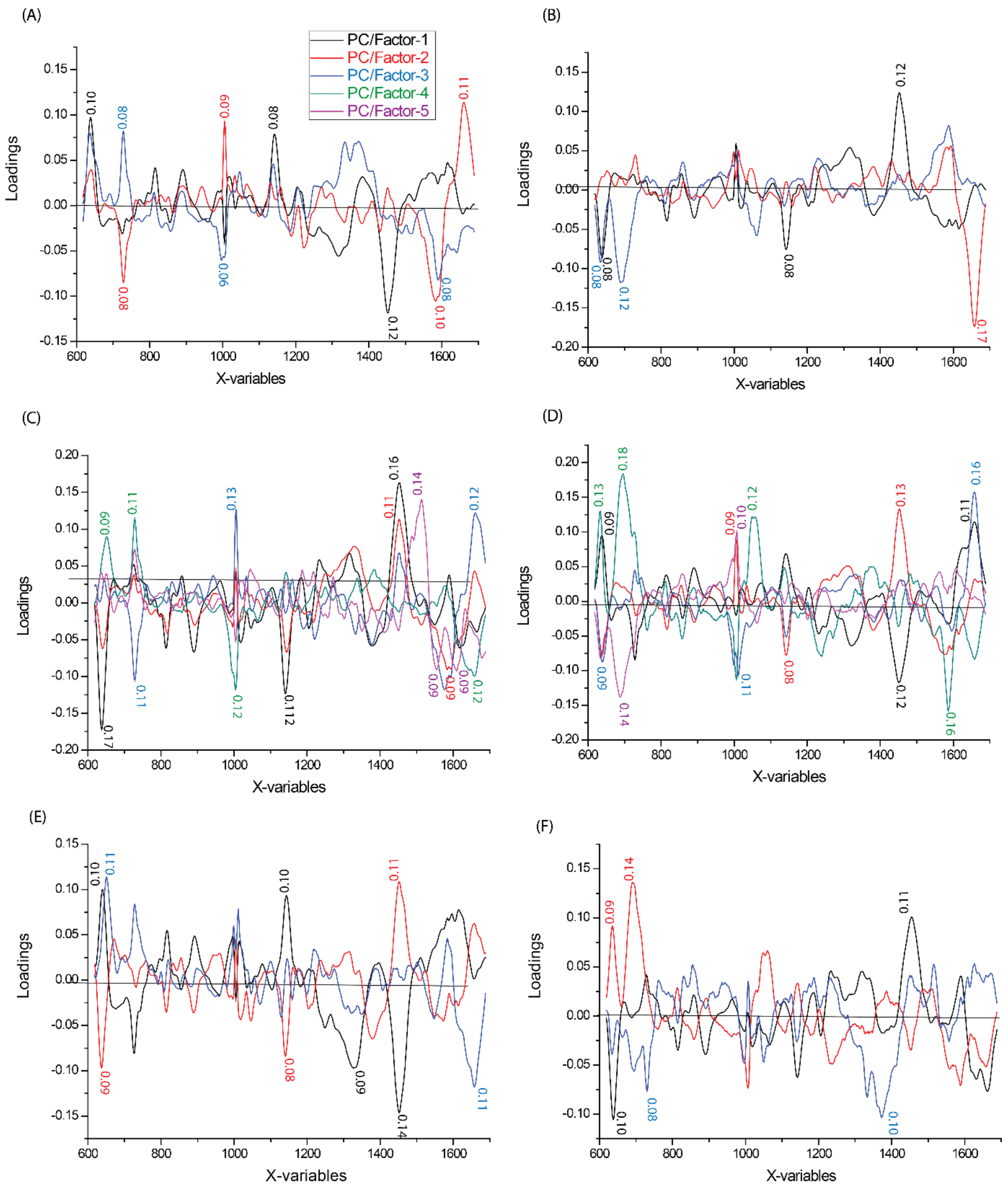

Figure S5. Loadings of the first three/five components in the fingerprint region with reference to the Fig. 2A (**A**), Fig. 2B (**B**), Fig. 2C (**C**), Fig. 2D (**D**), Fig. 3SA (**E**), Fig. 3SB (**F**).

Table S3. The first three or five components/factors their calculated weight (absolute values) obtained for each PCR and PSL model.

Type of Model	Type of Sample	PCs	x-Variables	Loadings
**PCR**	Non-cancer and cancer	PC-1	638	0.10
			1142	0.08
			1452	0.12
		PC-2	728	0.08
			1005	0.09
			1581	0.10
			1660	0.11
		PC-3	727	0.08
			995	0.06
			1589	0.08

**PCR**	Type of cancer	PC-1	638	0.08
			1142	0.08
			1452	0.12
		PC-2	1660	0.17
		3	637	0.08
			690	0.12
**PLS**	Non-cancer and cancer	Factor-1	638	0.17
			1140	0.12
			1452	0.16
		Factor-2	1451	0.11
			1582	0.09
		Factor-3	728	0.11
			1005	0.13
			1660	0.12
		Factor-4	651	0.09
			728	0.11
			1004	0.12
			1656	0.10
		Factor-5	1514	0.14
			1554	0.09
			1610	0.09
**PLS**	Type of cancer	Factor-1	639	0.09
			1452	0.12
			1657	0.11
		Factor-2	1005	0.09
			1143	0.08
			1452	0.13
		Factor-3	639	0.09
			1011	0.11
			1657	0.16
		Factor-4	634	0.13
			693	0.18
			1052	0.12
			1584	0.16
		Factor-5	686	0.14
			1004	0.10
**PLS**	Women- non-cancer and cancer	Factor-1	635	0.10
			1144	0.10
			1331	0.09
			1448	0.14
		Factor-2	627	0.09
			1123	0.08
			1430	0.11
		Factor-3	642	0.11
			1635	0.11
**PLS**	Men- non-cancer and cancer	Factor-1	634	0.10
			1450	0.11
		Factor-2	634	0.09
			692	0.14
		Factor-3	728	0.08
			1371	0.10

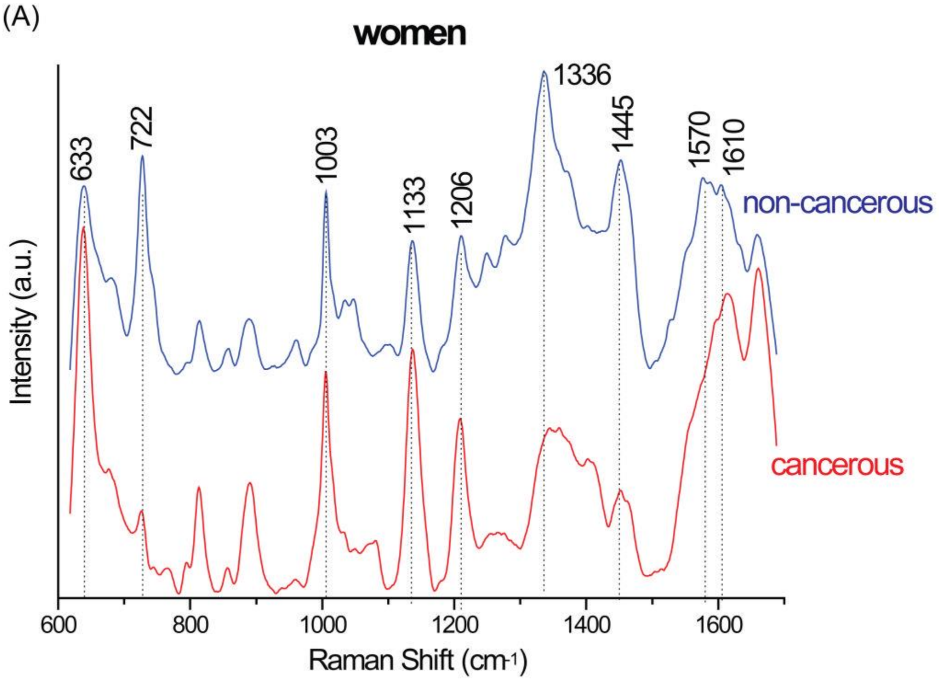

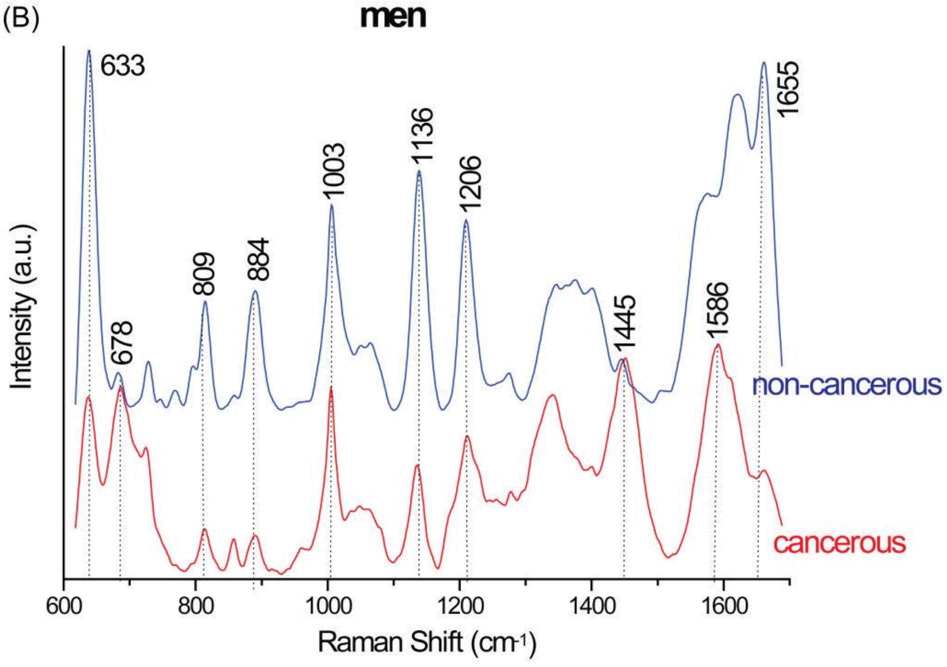

Figure S6. The averaged SERS spectra for non-cancerous and cancer samples for women (**A**) and for men (**B**).
